# Integrative Multi-Omics Analysis and Computational Modeling Identifying Shared Inflammatory Pathways and JAK Inhibitor Targets in PG and IBD

**DOI:** 10.3390/ijms27093733

**Published:** 2026-04-22

**Authors:** Hui Yao, Yi Wu, Ruzhi Zhang

**Affiliations:** Department of Dermatology, The Second Affiliated Hospital of Wannan Medical University, Wuhu 241000, China; huiyao0109@163.com (H.Y.); yiwu0227@163.com (Y.W.)

**Keywords:** pyoderma gangrenosum, inflammatory bowel disease, *JAK*-*STAT* signaling pathway, multi-omics integrated analysis, molecular docking, drug repurposing

## Abstract

This study investigates shared molecular mechanisms between pyoderma gangrenosum (PG) and inflammatory bowel disease (IBD) and systematically evaluates the therapeutic potential of *JAK* inhibitors targeting this pathway. Despite the clear clinical comorbidity, the core inflammatory pathways driving cross-tissue associations between the two diseases remain unclear. Furthermore, systematic mechanistic evidence is lacking regarding whether *JAK* inhibitors act by regulating shared pathological pathways in patients with comorbidities. To address this, this study integrated PG skin and IBD intestinal transcriptome data, single-cell transcriptomic data, and genome-wide association study (GWAS) meta-data from public databases. It employed a multi-level computational biology approach combining Mendelian randomization, weighted gene co-expression network analysis, protein interaction network construction, molecular docking simulations, and system dynamics modeling. The results revealed that genetic analysis confirmed IBD as a causal risk factor for PG, precisely identifying six shared genetic loci. Transcriptomic analysis identified a cross-tissue conserved inflammatory module centered on the *JAK*-*STAT* pathway, with *JAK2* and *STAT3* identified as network hubs. Molecular docking predicted high affinity of baricitinib for both *JAK1* and JAK2, while system dynamics modeling demonstrated that its intervention effectively suppresses signaling in the shared inflammatory network. This study reveals the molecular basis of the “gut–skin axis” comorbidity between PG and IBD from a multi-omics integration perspective. It provides predictive computational evidence for the use of *JAK* inhibitors in targeted comorbidity therapy. Baricitinib is identified as a particularly promising candidate. These findings advance the transition from empirical drug use to mechanism-guided precision treatment strategies. Although this study provides multiscale computational simulation evidence, the lack of direct experimental validation of these predicted results necessitates further confirmation through in vitro and in vivo experiments.

## 1. Introduction

Pyoderma gangrenosum (PG) is a rare, severe, and debilitating neutrophilic skin disorder. It is characterized by painful, rapidly progressing skin ulcers. These ulcers have violaceous margins and exhibit progressive creeping destruction. The condition is often accompanied by significant systemic involvement [1]. Diagnosis is challenging, primarily relying on clinical features and exclusion of other potential causes. The precise pathogenesis remains incompletely understood. However, it is widely believed to involve dysregulation of the innate immune system, neutrophil dysfunction, and excessive production of inflammatory cytokines [2]. Inflammatory bowel disease (IBD) is an umbrella term for chronic, recurrent inflammatory gastrointestinal disorders. It primarily comprises Crohn’s disease (CD) and ulcerative colitis (UC). The pathological basis involves abnormal immune responses of the intestinal mucosal immune system. These responses are directed against environmental factors such as the gut microbiota [3]. Although IBD lesions primarily affect the digestive tract, it frequently presents with multiple extraintestinal manifestations. Skin involvement is the most common. PG stands as one of the most representative severe dermatological complications [4]. Epidemiological studies indicate two key prevalence rates: approximately 30–50% of PG patients also have IBD, while about 1–5% of IBD patients develop PG. This comorbidity rate is significantly higher than in the general population. This observation strongly suggests an intrinsic biological link between the two diseases. The link transcends mere coincidence [5]. Clinically, patients with comorbidities often face more complex disease states. Skin lesions frequently fluctuate in parallel with intestinal disease activity. Treatment proves more challenging. This imposes significant physical and psychological distress, as well as economic burdens, on patients [6]. Therefore, delving into the shared pathophysiological basis of these two diseases offers two major benefits. First, it aids in revealing their fundamental nature. Second, it provides crucial scientific evidence for developing efficient, unified treatment strategies targeting comorbidity.

Currently, both PG and IBD treatments face significant limitations and unmet clinical needs. First-line therapies for PG typically involve topical or systemic corticosteroids and immunosuppressants such as cyclosporine and mycophenolate mofetil. However, these drugs carry pronounced side effects. Some patients exhibit poor response or intolerance [7]. For patients with comorbid IBD, treatment must address both skin and intestinal lesions simultaneously. Traditional medications like tumor necrosis factor-alpha inhibitors are effective for some patients. However, issues of primary or secondary failure persist. Additionally, there are potential risks such as the induction of infections [8]. IBD treatment has entered the era of biologics and targeted small-molecule drugs. Yet it similarly faces challenges. These include substantial therapeutic heterogeneity and insufficient long-term safety data [9]. In recent years, the Janus kinase-signaling pathway has garnered extensive attention for its role in various immune-mediated inflammatory diseases. This pathway serves as a central hub for cytokine signaling, regulating immune cell activation, differentiation, and function. Its abnormal activation is closely associated with the pathogenesis of multiple diseases, including rheumatoid arthritis, psoriasis, and IBD [10]. Inhibitors targeting this pathway, known as *JAK* inhibitors, have become important therapeutic weapons for treating moderate-to-severe rheumatoid arthritis, pyoderma gangrenosum, and UC [11]. Notably, case reports and small case series suggest that *JAK* inhibitors like tofacitinib and baricitinib may demonstrate potential therapeutic efficacy for refractory PG. This includes cases associated with IBD [12,13]. However, these observational findings lack in-depth mechanistic explanations. *JAK* inhibitors may exert their effects by regulating key shared pathological pathways between PG and IBD. This possibility remains to be elucidated through systematic research. Therefore, it is important to identify common molecular nodes between the two diseases. We must also rationally evaluate the potential of *JAK* inhibitors to target these nodes. This holds significant translational medical value.

Although clinical observations strongly support an association between PG and IBD, the core molecular mechanisms driving this comorbidity remain an unresolved scientific question. Previous studies have predominantly focused on clinical phenotype correlations or single pathway hypotheses. They lack a systematic, integrative perspective. This perspective would span from the genome to the proteome and from the population to the cellular level [14]. Hypotheses suggest a shared genetic background, dysregulated mucosal and skin barrier function, and common immune-inflammatory pathways may form their underlying basis [15]. For instance, genome-wide association studies have independently identified dozens of genetic risk loci associated with IBD [16]. Some of these loci involve innate immunity and interleukin signaling. However, genetic studies specifically targeting PG remain limited in scale. The extent of genetic overlap with IBD, along with specific shared loci, remains unclear [17]. At the transcriptomic level, studies have separately characterized gene expression profiles in PG skin lesions and IBD intestinal mucosa separately. However, research directly comparing the two is virtually absent [18,19]. Such a comparison is needed to identify cross-tissue conserved dysregulated gene modules. This cross-tissue conservation refers to the persistence of active gene programs across different organ pathologies. It likely points to core mechanisms driving systemic inflammation. Furthermore, cytokines such as tumor necrosis factor-α, interleukin-1β, interleukin-17, and interleukin-23 have been reported to be elevated in both diseases [20,21]. This suggests a potential shared cytokine network dysregulation. The *JAK*-*STAT* pathway serves as the primary downstream signal transmitter for these key cytokines. Does this pathway exhibit characteristic activation in the pathological tissues of both diseases? Are the key regulatory molecules upstream and downstream consistent? Answering these questions is crucial for two reasons. It helps us understand the mechanisms of comorbidity, and it allows for precisely identifying therapeutic targets.

It is worth emphasizing a key point. Genetic associations and transcriptional reprogramming constitute the core pillars of the comorbidity mechanism. Yet, a substantial body of circumstantial evidence suggests that epigenetic regulation and the gut microbiome may act as key regulatory layers. These layers jointly shape the inflammatory dialogue within the gut–skin axis. Alterations in DNA methylation patterns may weaken its negative feedback inhibitory function. For example, hypermethylation of the *SOCS3* gene promoter region can lead to sustained activation of the *JAK*-*STAT* pathway. Meanwhile, dynamic changes in histone acetylation modifications may affect the accessibility of *STAT3* target loci. This process thereby determines the transcriptional intensity of downstream effector molecules. On the other hand, gut microbiota dysbiosis causes shifts in functional metabolites. These shifts include decreased concentrations of short-chain fatty acids such as butyrate and valerate. Another shift is the redirection of tryptophan metabolism toward the kynurenine pathway. These metabolic changes may exert systemic effects on distal skin immune surveillance via the circulatory system. Consequently, this exacerbates neutrophILmediated skin damage. Current public databases lack matched epigenomic or multi-omic gut microbiota sequencing data for PG patients. This prevents this study from directly quantifying and integrating these regulatory layers. Nonetheless, incorporating them at the conceptual framework level is helpful. It situates the *JAK*-*STAT* core module within a more complex, multi-layered regulatory network. This network more closely mirrors in vivo conditions. The *JAK*-*STAT* module was identified by subsequent computational analyses. This theoretical framework also points to specific directions for future research aimed at expanding the omics dimensions.

High-throughput sequencing technologies have advanced rapidly, and public biomedical databases have grown increasingly abundant. As a result, computational biology approaches now enable the in-depth exploration of disease mechanisms without conducting new experiments [22]. Bioinformatics, particularly multi-omics data integration analysis, provides powerful tools. These tools help decipher the molecular networks underlying complex diseases at the systems level [23]. Genomic, transcriptomic, and protein interaction data can be integrated. From these data, disease-related molecular networks can be constructed. The resulting networks help identify key driver genes and pathway modules [24]. Molecular docking simulations, based on the three-dimensional structures of drugs and target proteins. They can predict binding patterns and affinities at the atomic level. This provides theoretical support for repurposing existing drugs or optimizing drug design [25]. This study aims to systematically investigate shared pathological mechanisms between PG and IBD using these computational methods. It also aims to rationally evaluate the therapeutic potential of *JAK* inhibitors. The research will first utilize publicly available genome-wide association study (GWAS) meta-data and transcriptomic datasets. This step will validate the intrinsic link between the two diseases. The validation will be performed at two levels: genetic association and gene expression. Subsequently, weighted gene co-expression network analysis will be performed. This analysis will identify core gene modules highly associated with both diseases within the transcriptome data. These modules will undergo multi-level bioinformatics analysis. This analysis includes pathway enrichment, protein interaction network construction, and hub gene screening. The goal is to pinpoint key shared inflammatory pathways and core targets. Finally, this study will combine molecular docking simulations and network pharmacology analysis. It will predict interactions between existing *JAK* inhibitors and core targets. This study will also simulate the impact of drug intervention on the shared inflammatory network at the systems level. This simulation will provide forward-looking computational evidence. It will also offer a theoretical framework for the application of *JAK* inhibitors as targeted therapies for PG and IBD comorbidity.

This study employs a multi-omics integrated bioinformatics strategy to address the clinical challenge of PG and IBD comorbidity. Through systematic analysis, it is anticipated that conserved molecular pathways linking skin and intestinal inflammation will be revealed. In particular, this analysis clarifies the central role of the *JAK*-*STAT* pathway. Furthermore, it provides robust computational mechanisms and quantitative evidence to support the repurposing or prioritization of *JAK* inhibitors such as baricitinib and tofacitinib. The findings are expected to transcend traditional single-disease research paradigms, offering novel insights to advance the precision medicine concept of “one drug treating multiple comorbidities” by targeting disease comorbidity mechanisms. This work lays a robust theoretical foundation for subsequent translational research and clinical trial design.

## 2. Results

### 2.1. Transcriptomic Commonality Between PG and IBD

**Figure 1:**
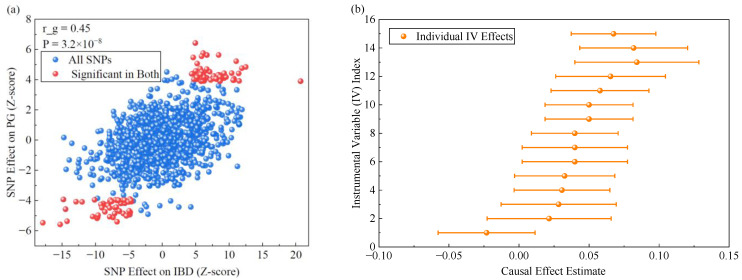

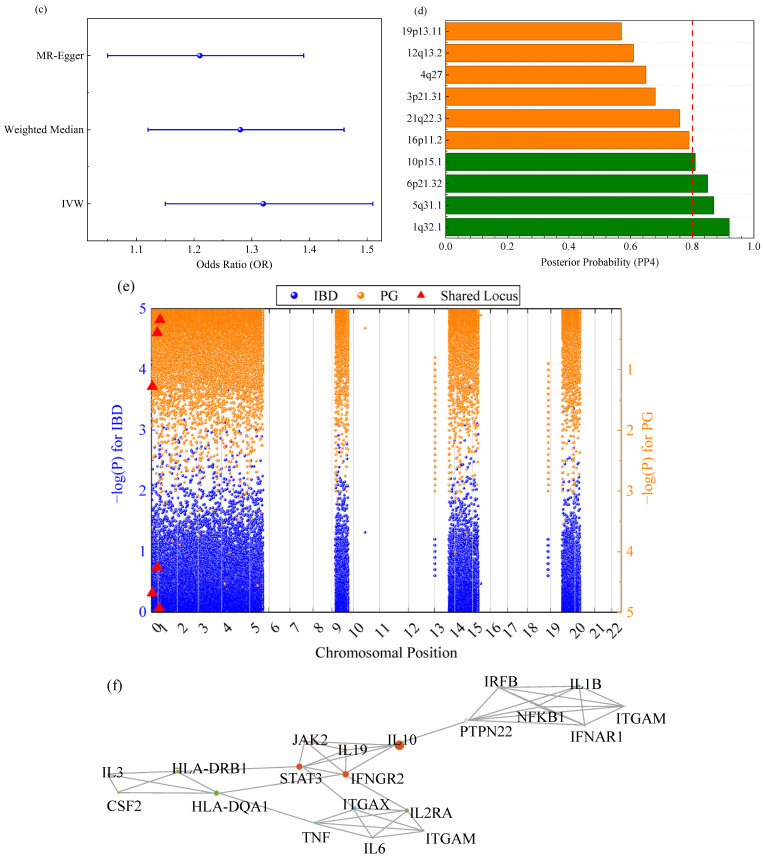
Genetic Structure of PG and IBD: (**a**) genetic correlation scatter; (**b**) effect sizes and confidence intervals of primary instrumental variables; (**c**) point estimates and confidence intervals from three Mendelian randomization methods; (**d**) top 10 genomic regions ranked by posterior probability; (**e**) upper panel shows genome-wide association study *p*-values for IBD, lower panel shows *p*-values for PG; red markers indicate six high-probability shared loci identified by colocalization analysis; (**f**) protein interaction network of candidate genes.

To investigate the genetic link between pyoderma gangrenosum and inflammatory bowel disease (IBD), we first conducted genetic association analysis and causal inference. Using pooled data from large-scale genome-wide association studies, we assessed the overall genetic correlation between the two diseases. Calculations were based on the linkage disequilibrium (LD) score regression method, which estimates genetic correlations between traits by examining the relationship between the magnitude of single-nucleotide polymorphism effects and their linkage disequilibrium structures. The genetic correlation scatterplot is shown in Figure 1a. The analysis revealed a significant positive genetic correlation between PG and IBD, particularly with CD subtypes. The estimated correlation coefficient was 0.45. The standard error was 0.08. The significance *p*-value was 3.2 × 10^−8^. This finding indicates substantial shared genetic risk factors between the two diseases, partially explaining their clinical comorbidity. The distribution pattern of points in Figure 1a aligns with a significant non-zero correlation coefficient, providing intuitive confirmation of genetic overlap.

After confirming a significant genetic association, a Mendelian randomization (MR) analysis framework was employed to further infer potential causal relationships between the two conditions. Inverse variance weighting served as the primary analysis method, supplemented by weighted median weighting and MR-Egger regression for sensitivity testing to assess pleiotropy bias. The study aimed to further ensure the robustness of causal inference against violations of horizontal pleiotropy. For this purpose, three additional sensitivity analyses were performed. First, Cochran’s Q statistic was calculated to quantify heterogeneity among instrument-specific causal estimates. A significant Q value may indicate pleiotropic effects. Second, the MR-PRESSO (Mendelian Randomization Pleiotropy RESidual Sum and Outlier) global test was applied to detect and correct for horizontal pleiotropy via identification and removal of outlier genetic variants. Third, a leave-one-out analysis was conducted. We sequentially excluded each genetic variant from the instrumental variable set. We then re-estimated the causal effect. The goal was to assess whether any single variant disproportionately influenced the overall estimate. The analysis identified 127 high-quality genetic instrumental variables strongly and independently associated with IBD. Among these, the top 15 were designated primary instruments; their effect sizes and confidence intervals are shown in Figure 1b. Comparisons of point estimates and confidence intervals across the three Mendelian randomization methods are shown in Figure 1c. As evident from Figure 1c, genetically predicted increased IBD risk is a causal risk factor for PG development, with an odds ratio of 1.32, a 95% confidence interval ranging from 1.15 to 1.51, and a *p*-value of 7.1 × 10^−5^. Sensitivity analysis results aligned with the main analysis direction: the weighted median method estimated an OR of 1.28, while the MR-Egger regression yielded an OR of 1.21. The MR-Egger regression intercept *p*-value exceeded 0.05, indicating no significant pleiotropy. Additional sensitivity analyses further substantiated the absence of substantial horizontal pleiotropy. The Cochran’s Q test revealed no significant heterogeneity among the 127 instrumental variables (Q = 142.3, *p* = 0.146). The MR-PRESSO global test did not detect evidence of horizontal pleiotropy (*p* = 0.281), and no outlier variants were identified for correction. Leave-one-out analysis demonstrated a stable and directionally consistent causal estimate. This consistency was observed after the sequential removal of each individual SNP. All leave-one-out OR estimates fell within the 95% confidence interval of the primary inverse variance weighted estimate. The consistency between Figure 1b,c enhances the reliability of causal inference.

To precisely localize shared genetic signals within specific genomic regions, Bayesian co-localization analysis was performed on genome-wide association signals for PG and IBD. The posterior probability distribution from co-localization analysis is shown in Figure 1d. The study focused on known IBD risk loci across the genome. It calculated the posterior probability of a single shared causal variant at each locus. The co-localization analysis identified six genomic regions with high posterior probabilities. Among them, the locus on chromosome 1q32.1 exhibited the highest probability of shared causal variation at 0.92. This region contains known immune-related genes. Other significant regions involve genes associated with the interleukin signaling pathway and intestinal barrier function.

To further visualize these shared genetic loci, a cross-trait Manhattan plot was constructed. This plot integrates significant *p*-values from genome-wide association studies of PG and IBD onto a single genomic coordinate. It highlights shared risk loci confirmed by co-localization analysis. The cross-trait Manhattan plot is shown in Figure 1e. The figure reveals that multiple loci reaching genome-wide significance in IBD also exhibit significant association signals in PG. These findings provide genetic anchors for understanding shared pathophysiological pathways between the two diseases.

To elucidate the potential biological functions of candidate genes within shared loci, protein interaction networks were constructed for the six high-probability co-localization regions identified above. Network nodes represent genes, while edges denote known or predicted protein–protein interactions. Network analysis revealed significant enrichment of these genes in several key immune-related pathways, including the *JAK*-*STAT* signaling pathway, tumor necrosis factor signaling pathway, and cytokine-cytokine receptor interaction pathway. Multiple genes occupied central hub positions within the network. This suggests that they may play pivotal roles in regulating shared immune dysregulation. The protein interaction network of candidate genes is depicted in Figure 1f. This network diagram visually demonstrates that genes pointed to by shared genetic loci do not exist in isolation. Instead, they form a tightly interacting molecular functional module, collectively pointing to the core mechanism of dysregulated immune responses.

Table 1 further details the genomic coordinates, most significant single-nucleotide polymorphisms, association *p*-values, posterior probabilities, and annotated key candidate genes within the six major shared loci identified by colocalization analysis. This information provides prioritized genetic clues for subsequent functional studies and drug target screening. Multi-level evidence, from population genetics to precise genomic localization, consistently demonstrates a substantial biological association between PG and IBD. This association is driven by shared genetic risk variants. This common genetic basis likely constitutes the fundamental cause of comorbidity between the two diseases. It does so by influencing key immunoregulatory pathways. This insight points the way toward identifying shared therapeutic targets.

To further elucidate the potential biological functions of the *IL10* and *IL19* genes within the 1q32.1 co-localization region in the gut–skin axis at the cellular level, this study utilized single-cell transcriptomic data. The data were used to evaluate the cell-type-specific expression profiles of these two genes in PG lesions and IBD intestinal mucosa. In the single-cell transcriptome of PG lesions, both *IL10* and *IL19* exhibited highly consistent patterns of enrichment in myeloid cells. Their transcriptional signals were primarily concentrated in two subpopulations: those annotated as *CD163*-positive macrophages and *CD1C*-positive dendritic cells. In contrast, expression was virtually undetectable in keratinocytes or fibroblasts. Quantitative analysis revealed that the mean log-normalized expression value of *IL10* in the myeloid cell population was 1.52, with 34.7% of cells expressing the gene. The mean log-normalized expression value of *IL19* was 1.28, with 28.9% of cells expressing the gene. The expression levels of both genes in myeloid cells were significantly higher than in other cell types; all comparisons yielded *p*-values < 0.001 according to the Wilcoxon test. In independent single-cell data from IBD intestinal mucosa, high expression of *IL10* and *IL19* was similarly observed in myeloid cells. These myeloid cells included inflammatory macrophages and activated dendritic cells. In contrast, signals within intestinal epithelial cells were very weak. This expression pattern is conserved across skin and intestinal tissues, yet it is restricted to myeloid immune cell populations. This suggests that the shared genetic locus may influence inflammatory processes in both organs primarily by regulating the function of innate immune cells, particularly macrophages and dendritic cells. *IL10* is a core immunoregulatory factor. Its myeloid-derived production is critical for maintaining tissue immune homeostasis. *IL19* promotes the differentiation of macrophages toward a pro-inflammatory phenotype via an autocrine loop. Given these roles, this cell-type-specific expression distribution provides key functional insights. It explains how genetic variants at the 1q32.1 locus simultaneously increase susceptibility to both PG and IBD. Moreover, it reinforces the immunological basis of the gut–skin axis comorbidity hypothesis at single-cell resolution.

### 2.2. Cross-Tissue Regulatory Network Analysis of Inflammatory Pathways

**Figure 2:**
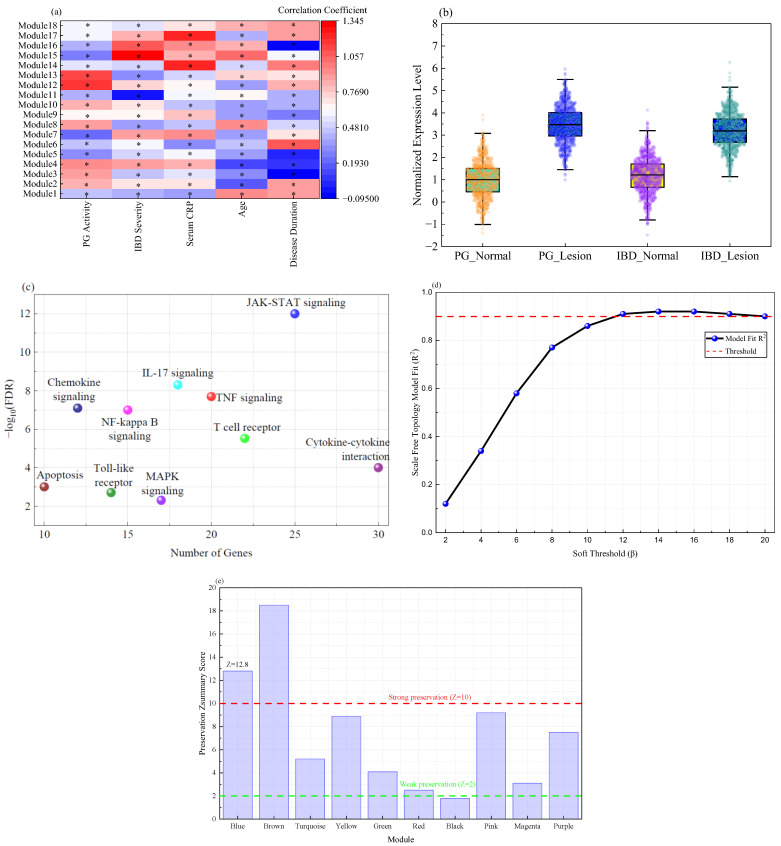
Module and Pathway Analysis: (**a**) heatmap of module-clinical trait association analysis, * represent 10% significance level; (**b**) gene expression patterns; (**c**) pathway enrichment bubbles; (**d**) scale-free topology model fitting index R^2^ across a range of soft threshold powers from 2 to 20. The red horizontal line indicates the threshold of 0.90. (**e**) Module preservation Zsummary statistics comparing the preservation of all identified modules between the PG skin dataset and the IBD intestinal dataset. The dashed red line indicates the threshold for strong preservation, Z = 10.

To systematically elucidate the core regulatory networks driving the comorbidity of PG and IBD, the study first mined batch-corrected the merged transcriptomic data using weighted gene co-expression network analysis. The ComBat-adjusted expression matrix successfully removed the dominant source of technical variation attributable to dataset origin. This was evidenced by principal component analysis. In this analysis, the first two principal components separated samples predominantly by disease status rather than by study accession. By setting the soft threshold power to 12, a scale-free topological network was successfully constructed, identifying 18 highly co-expressed gene modules. The correlation analysis between modules and clinical traits revealed that Module 4 exhibited extremely significant positive correlations with disease activity in both conditions. The correlation coefficients were 0.92 and 0.88, respectively. This module also showed a strong association with serum C-reactive protein levels. Specific correlation strengths are depicted in Figure 2a. This study designates Module 4 as the Blue Module. The heatmap visually presents the association matrix between different modules and a series of clinical-pathological features. The Blue Module demonstrates a unique indicative value for inflammatory states.

Further analysis of the expression patterns of 325 genes within the blue module is shown in Figure 2b. This subplot depicts the expression profiles of module-characteristic genes across different sample groups. It clearly demonstrates that these genes are synchronously and significantly upregulated in PG and IBD lesion tissues. Conversely, they are generally downregulated in their respective healthy control tissues. This confirms the cross-tissue comorbidity association of this module at the transcriptional level. Kyoto Encyclopedia of Genes and Genomes (KEGG) pathway enrichment analysis of the blue module is summarized in Figure 2c as a bubble plot. The module genes show significant enrichment in classical inflammatory and immune regulatory pathways, including *JAK*-*STAT* signaling, *IL17* signaling, and *TNF* signaling pathways. The *JAK*-*STAT* pathway exhibits the highest enrichment significance, suggesting its pivotal role as a central hub in the comorbidity mechanism. The results of the sensitivity analysis are shown in Figure 2d,e. These confirm the robustness of the parameters. The scale-free topology fitting index *R*^2^ was computed for a range of candidate powers from 2 to 20. The power value of 12 was confirmed as the point where the fitting curve first reaches a plateau with an *R*^2^ value exceeding 0.90. Therefore, it satisfies the criterion for a scale-free network structure. Subsequently, the preservation of the clinically significant module, designated the blue module, was evaluated across a series of alternative soft threshold settings. These settings were specifically *β* = 8, *β* = 10, and *β* = 14. Module eigengene values and gene membership assignments for the blue module exhibited high correlation coefficients greater than 0.95 across all tested powers. This indicates that the composition and representative expression profile of the module remain highly stable. Furthermore, to address the cross-tissue nature of our analysis, a module preservation analysis was conducted between the skin-derived dataset and the intestinal-derived dataset. This was done using the modulePreservation function with 200 permutations. This analysis yielded a Zsummary preservation score of 12.8 for the blue module. This score falls well within the range defined as strong preservation (Zsummary greater than 10). These findings confirm that the identified transcriptional module is not an artifact of a specific parameter choice. It is robustly conserved across the two distinct tissue environments.

**Figure 3:**
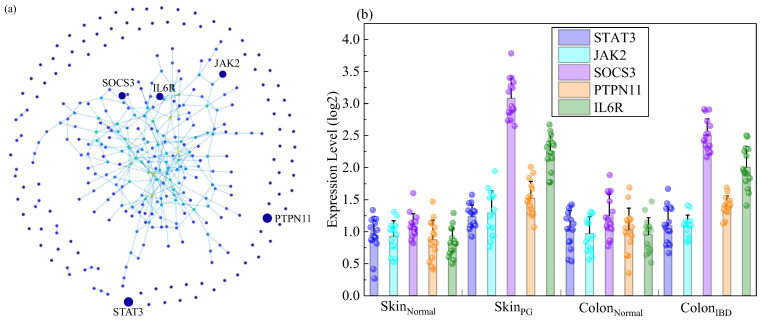
PPI Network and Hub Genes: (**a**) PPI network diagram; (**b**) hub gene expression profile.

To decipher the regulatory hub within the blue module, To achieve this, this study constructed a protein interaction network for its genes based on the STRING database. As shown in Figure 3a, this network diagram comprises over 300 nodes and 1500 interaction edges. The size and color intensity of each node represent its degree centrality. Several key nodes occupying central positions within the network were identified, including *JAK2*, *STAT3*, *SOCS3*, *IL6R*, and *PTPN11*. These highly central genes functionally cluster around cytokine-mediated signal transduction and immune response processes. This study further mapped the expression profiles of these hub genes across different tissues. This validation aimed to confirm expression alterations under disease conditions, as shown in Figure 3b. This subgraph reveals a consistent trend. Genes including *STAT3* and *JAK2* are consistently upregulated in pathological tissues of both diseases. This upregulation is evident when compared to normal skin and intestinal tissues. In contrast, the expression pattern of the negative feedback regulator SOCS3 exhibits greater complexity, suggesting its potential involvement in regulating drug response mechanisms. These core hub genes are further detailed in Table 2, which presents their degree centrality, betweenness centrality, major enriched KEGG pathways, and associated biological processes.

### 2.3. Prediction of JAK Inhibitor Targeting

Based on the previously identified shared inflammatory pathways between PG and IBD, this study performed a systematic prediction of *JAK* inhibitors targeting to translate theoretical mechanisms into precision treatment strategies. This analysis followed a multi-level computational framework. It sequentially incorporated topological screening of key target networks, molecular docking simulations of drug-target interactions, and systematic modeling of signaling pathway dynamics under drug intervention. The aim was to evaluate the therapeutic potential of *JAK* inhibitors from both static interaction and dynamic regulation dimensions.

**Figure 4:**
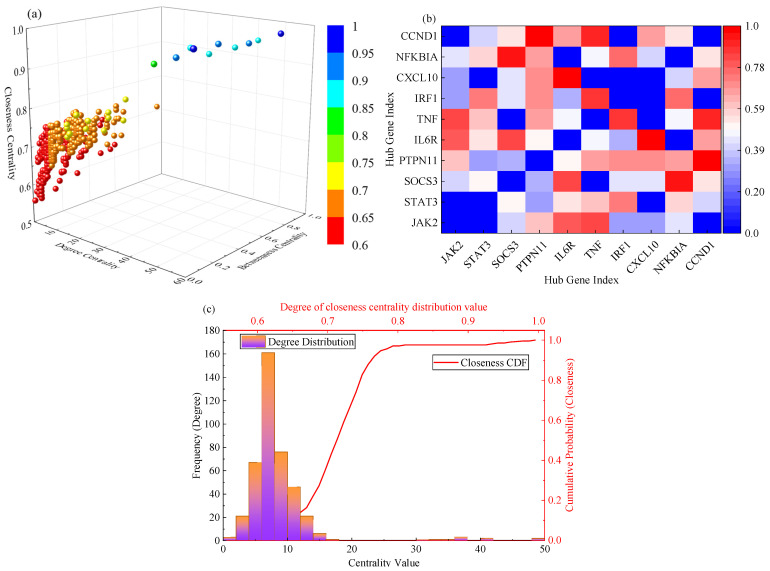
Global Features and Core Subnetworks of the Network Structure: (**a**) 3D Network Centrality Scatter Plot; (**b**) Hub Gene Interaction Strength Matrix; (**c**) Centrality Distributions.

To identify core targets with the highest regulatory value among shared differentially expressed genes. To do this, this study constructed a protein interaction network that included all 412 shared genes. Topological analysis of this network identified the top 10 high-order nodes as core hub genes. These genes play a decisive role in maintaining network connectivity and signaling efficiency. The global features of the network structure and the core subnetwork are illustrated in Figure 4. Figure 4a shows a three-dimensional scatter plot depicting the distribution of all network nodes across degree centrality, betweenness centrality, and closeness centrality. Most nodes cluster near the origin. In contrast, a few nodes deviate significantly from the center. These nodes exhibit high values across all three centrality metrics. This visually confirms their pivotal and bridging roles within the network. Figure 4b further focuses on the interactions among these 10 core hub genes. Their connection strength matrix reveals a highly dense internal connectivity pattern, indicating that these genes do not act in isolation but form a functionally highly synergistic regulatory module. Figure 4c shows the statistical distribution characteristics of degree centrality and closeness centrality across the entire network through distribution histograms and cumulative distribution curves, further validating the rarity and distinctiveness of core hub genes at the population level.

Table 3 details the quantitative topological metrics and biological functional annotations for these 10 core hub genes. These data reveal that *JAK2* and *STAT3* rank at the top in degree centrality, betweenness centrality, and closeness centrality. This finding highlights their absolute core status within the signaling network. Notably, *SOCS3*—a key negative feedback regulator in the *JAK*-*STAT* pathway—is also identified as a core node. Its identification suggests that targeting this node may be crucial for restoring immune homeostasis. Together, these genes form a complete signaling axis spanning from cell membrane receptors, intracellular kinases, and transcription factors to downstream effector molecules, providing clear targets for multi-target drug interventions.

**Figure 5:**
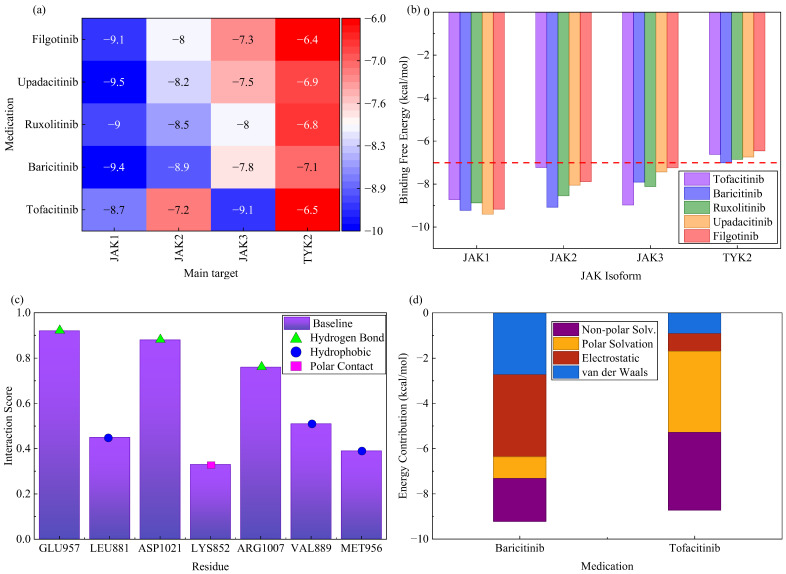
Molecular Docking Binding Energy Calculation Results: (**a**) Binding Energy Heatmap; (**b**) Energy by Isoform; (**c**) Baricitinib-*JAK1* Binding Pocket; (**d**) Energy Decomposition Comparison.

To evaluate the binding efficacy of existing clinical *JAK* inhibitors with the aforementioned core targets, particularly *JAK* family kinases, systematic molecular docking simulations were conducted. Five drugs—tofacitinib, baricitinib, ruxolitinib, upadacitinib, and filgotinib—were selected for docking against the active sites of *JAK1*, *JAK2*, *JAK3*, and *TYK2*, respectively. The simulation results are presented in multiple visual formats in Figure 5. Figure 5a provides an intuitive heatmap display of the predicted binding free energies for all drug-target pairs. In this heatmap, color intensity represents binding strength. The figure clearly shows that all drugs exhibit strong binding potential for both *JAK1* and *JAK2*. Among them, baricitinib and upadacitinib display the lowest predicted binding energies for *JAK1*. These quantitative results confirm that baricitinib and upadacitinib exhibit the strongest predicted binding affinities for *JAK1*. Baricitinib also demonstrates outstanding binding capacity for *JAK2*. Tofacitinib exhibits higher selectivity for *JAK3*. These computational biophysical data provide theoretical support for the target preference of different *JAK* inhibitors. The findings suggest that baricitinib may possess the dual advantage of simultaneously and efficiently inhibiting both *JAK1* and *JAK2*. Figure 5b further compares binding energy differences across *JAK* subtypes using grouped bar charts. The commonly used activity threshold of −7.0 kcal/mol is indicated in the chart. All drugs exhibit binding energies superior to this threshold for both *JAK1* and *JAK2*. Figure 5c details key interactions within the binding pocket of baricitinib with JAK1, including hydrogen bonds and hydrophobic interactions formed with residues such as *GLU957* and *ASP1021*, structurally explaining its high affinity. Figure 5c also shows the predicted binding conformation of baricitinib within the *JAK1* ATP-binding pocket. In this conformation, the pyrimidine core forms critical hydrogen bonds with the hinge region residues *GLU957* and *LEU959*. The ethylsulfonyl terminus extends into the anterior pocket defined by *ASP1021* and *ARG1007*. This conformation is consistent with the binding mode of classic Type I kinase inhibitors. This consistency confirms that the docking simulation accurately identified the competitive interactions targeting the ATP-binding site. Figure 5d compares the energy contribution to the binding of baricitinib and tofacitinib to *JAK1* via stacked bar charts, revealing van der Waals forces as the primary driver while highlighting differences in electrostatic contributions between the drugs.

**Figure 6:**
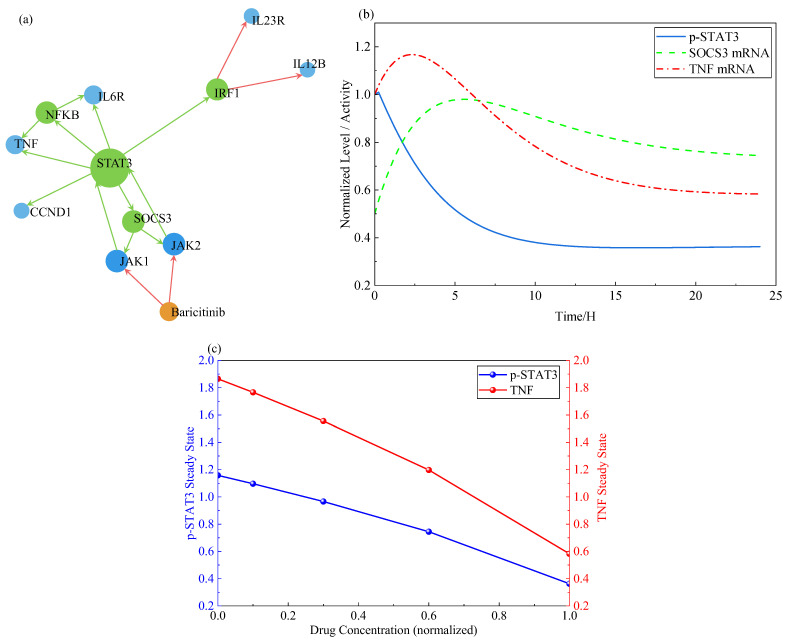
Drug-perturbation network and kinetic simulation results: (**a**) *JAK* Inhibitor Signaling Network; (**b**) Signaling Dynamics Post-Treatment; (**c**) Dose–Response Relationships.

To dynamically evaluate the impact of *JAK* inhibitor interventions on the aforementioned shared inflammatory network at the systems level, an integrated drug-perturbation network was constructed and subjected to kinetic simulations. Figure 6a depicts an expanded drug-regulation network that incorporates not only core members of the *JAK*-*STAT* axis but also integrates key downstream transcription factors such as *NF*-*κB* and *IRF1*, along with their target genes. The figure clearly illustrates how *JAK* inhibitor nodes, represented by baricitinib, directly act on *JAK1*/*JAK2*, subsequently cascading inhibitory signals through nodes like *STAT3* to the entire inflammatory network. Based on this network topology, Figure 6b presents simulated kinetic profiles of key signaling molecules following drug intervention. Simulation results indicate that following drug administration at time zero, activated *STAT3* protein levels rapidly decline, accompanied by a corresponding decrease in simulated expression levels of its downstream effector molecule *TNF*. In contrast, transcription levels of the negative feedback factor *SOCS3* exhibit a transient increase followed by a gradual decline, consistent with known biological feedback mechanisms. Simulated *SOCS3* transcript levels exhibited a transient surge within the first four hours after virtual dosing. This surge reflects an initial compensatory transcriptional activation in response to the abrupt blockade of the pathway. Subsequently, a progressive decline toward baseline occurred. This decline resulted from sustained suppression of phosphorylated *STAT3* below a critical threshold, which diminished the driving force for *SOCS3* transcription. This kinetic trajectory leads to the following prediction: Restoration of *SOCS3*-mediated homeostasis requires persistent and potent inhibition of *JAK1* and *JAK2* nodes. Such inhibition sufficiently depletes activated *STAT3* pools, thereby allowing the negative feedback loop to reset to a physiologic set point rather than remaining locked in a futile counter-regulatory overdrive.

To explicitly address the divergent selectivity of baricitinib and tofacitinib, the model parameters were adjusted to reflect their distinct *JAK* subtype preferences. Baricitinib was modeled with high-affinity inhibitory constants for *JAK1* and *JAK2*, *k_i_* values of 0.8 nM and 1.2 nM, respectively, while maintaining a substantially lower affinity for JAK3, with a *k_i_* value of 45 nM. In contrast, tofacitinib was parameterized with a primary high affinity for *JAK3*, a *k_i_* value of 1.5 nM, and lower affinities for *JAK1* and *JAK2*, *k_i_* values of 12 nM and 25 nM, respectively. Under these differential parameter settings, the simulated trajectories in Figure 6b correspond to the baricitinib regimen, revealing a rapid and sustained suppression of phosphorylated *STAT3* exceeding 75% reduction within two hours and a prolonged inhibition of *TNF* expression. When the simulation was repeated using the tofacitinib parameter set, the kinetic profile exhibited a more moderate and delayed reduction in p-STAT3 levels. Maximum inhibition reached only approximately 45% relative to baseline, and a noticeable recovery trend began after 8 h. This discrepancy in dynamic response is attributable to the preferential blockade of *JAK1* and *JAK2* by baricitinib, which directly attenuates *STAT3* activation upstream. In contrast, the *JAK3*-biased inhibition of tofacitinib leaves *JAK1* and *JAK2* partially active, thereby allowing residual signal transduction through alternative cytokine receptors. Consequently, the kinetic simulation provides a mechanistic explanation for how baricitinib may confer a more comprehensive suppression of the shared inflammatory network in the context of pyoderma gangrenosum and inflammatory bowel disease comorbidity. The dose–response curve in Figure 6c further reveals the relationship between drug efficacy and concentration, indicating that the steady-state inhibition levels of both p-*STAT3* and *TNF* increase with rising drug concentration, though not in a strictly linear fashion. This provides computational reference for optimizing clinical dosing.

This computational framework does not explicitly model thromboembolic risk. This risk is a safety signal associated with *JAK* inhibition in susceptible populations. Furthermore, the predicted binding profiles should not be extrapolated to infer clinical hazard without dedicated pharmacovigilance data integration. This study integrates network topology analysis, molecular docking, and system dynamics simulations. The results reveal that baricitinib demonstrates dual potential to efficiently inhibit both *JAK1* and *JAK2* in predictive models. Its intervention effectively suppresses core signaling pathways within shared inflammatory networks. These computational findings provide forward-looking evidence and mechanistic hypotheses for applying *JAK* inhibitors—particularly baricitinib—as targeted therapies for the comorbidity of PG and IBD.

### 2.4. Assessment of the Reproducibility of Key Findings in Independent Cohorts

**Figure 7:**
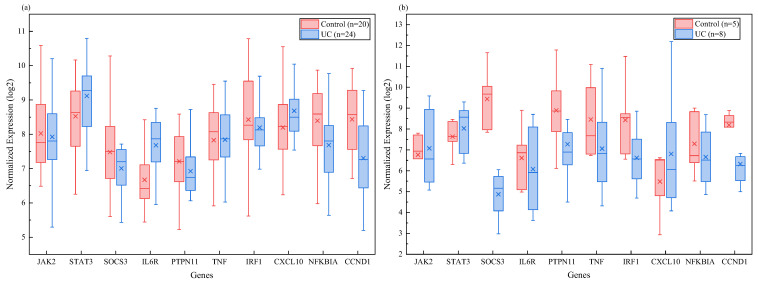
Expression levels of ten core hub genes in independent validation cohorts: (**a**) Gut Validation Cohort (GSE16879); (**b**) Skin Validation Cohort (GSE125734).

This study aimed to validate the external reproducibility of the tissue-conservative inflammatory modules and their hub genes identified in the main analysis. To this end, meta-validation was conducted in two transcriptomic data cohorts independent of the training set. Gene set analysis was performed in the UC mucosal validation cohort GSE16879. This analysis revealed that the overall enrichment score of the characteristic gene set of the blue module was significantly higher in the disease group than in the control group. The standardized enrichment score difference was 1.78. Additionally, the permutation test *p*-value was less than 0.001. These findings indicate that this co-expression module was also highly activated in another batch of IBD intestinal mucosal samples. The skin validation cohort GSE125734 was also examined. Although the sample size was smaller, the gene set variation analysis score for the blue module still showed a robust upward trend in PG lesions. The difference was 1.42, and the permutation test *p*-value was 0.008. The independent validation expression patterns of the ten core hub genes are shown in Figure 7. Among these, four genes—*JAK2*, *STAT3*, *TNF*, and *CXCL10*—showed consistent and statistically significant upregulation in lesion tissues from both the intestinal and skin validation cohorts. In contrast, the expression trend of *SOCS3* in the validation cohorts aligned with the complex expression pattern observed in the training set. Specifically, *SOCS3* exhibited feedback-mediated upregulation in some samples and relative suppression in others. This pattern is consistent with its biological role as a negative feedback regulator. Further correlation analysis indicated a high positive correlation between the log2 fold change vectors of each gene across the cohorts. Specifically, the Spearman correlation coefficient was 0.86 for the intestinal validation cohort and 0.79 for the skin validation cohort when compared to the original training set. Both coefficients reached statistical significance. Table 4 lists the differential expression statistics for the ten core genes across three datasets. These datasets include the training set (GSE107499 combined with GSE75214), the gut validation set (GSE16879), and the skin validation set (GSE125734). The table provides log2 fold changes and adjusted *p*-values for direct comparison. These results confirm that the *JAK*-*STAT* inflammatory axis signature identified in this study is not due to biases introduced by specific datasets or technical platforms, but rather exhibits robustness across sample sources and sequencing strategies.

**Figure 8:**
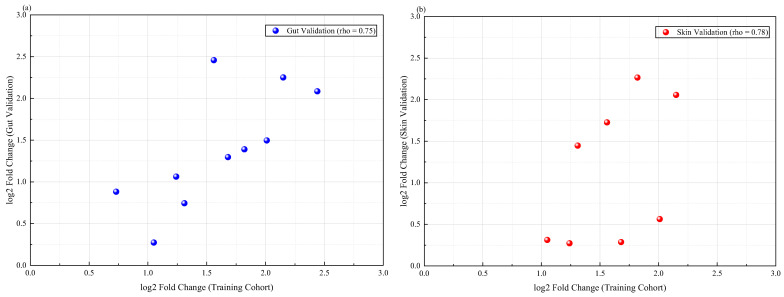
Correlation of log2 fold changes between training and validation cohorts: (**a**) Gut Validation; (**b**) Skin Validation.

To further obtain external validation from the published literature, this study systematically compared the aforementioned ten core hub genes with lists of key molecules identified in existing transcriptomic and proteomic studies of PG or IBD [26,27,28,29,30]. The results of this comparison are shown in Figure 8. The comparison revealed that *JAK2* and *STAT3* were reported in all five studies as differentially expressed or as key nodes upstream of core signaling pathways. *TNF*, *IRF1*, and *CXCL10* were frequently mentioned, while *SOCS3* and *NFKBIA* were identified as potential feedback regulatory checkpoints. Such a high degree of overlap provides robust literature-based external validation for the network analysis results of this study. This indicates that the targets identified by the computational workflow are not isolated findings. Instead, they are consistent with the conclusions drawn from converging evidence across multiple sources in the current field.

### 2.5. Stratification by Disease Subtypes and Heterogeneity in Pathway Activity Reveal Clues to Individual Differences

**Figure 9:**
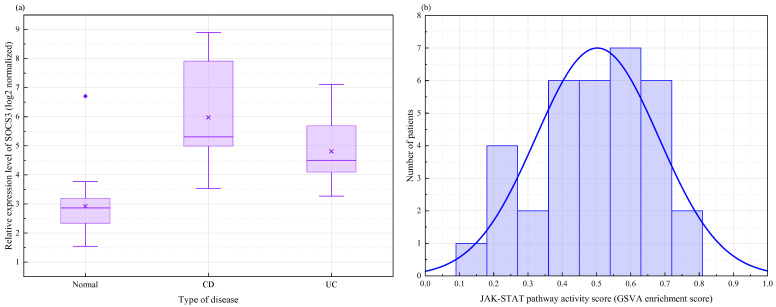
Subtype-based stratification analysis and assessment of inter-patient heterogeneity in pathway activity: (**a**) comparison of *SOCS3* expression across different disease subtypes; (**b**) inter-patient distribution of *JAK*-*STAT* pathway activity scores in patients with intestinal lesions.

Building on the findings regarding population-wide average levels, this study further investigated potential molecular heterogeneity within the patient population. Using the well-defined disease subtype annotations in the GSE75214 dataset, we performed a subtype-stratified analysis of the blue module. This analysis focused on overall expression levels and core hub genes for CD and UC, as shown in Figure 9a. Analysis of gene set variations revealed that the overall enrichment scores of the blue module were significantly elevated in both disease groups compared to the healthy control group. The standardized enrichment score differences were 1.92 for CD and 1.85 for UC. Permutation test *p*-values were less than 0.001 for both comparisons. The direct comparison between the two subtypes did not reach the threshold for statistical significance. However, the median module score in the CD group was slightly higher than that in the UC group. This suggests that subtle differences in severity or regulatory mechanisms may exist against a shared inflammatory background. Individual analysis of the ten core hub genes revealed a more nuanced picture. *JAK2*, *STAT3*, and *TNF* exhibited consistent strong upregulation in both subtypes with no significant intergroup differences. This further confirms the status of the *JAK*-*STAT* axis as a shared core mechanism. However, the upregulation of the negative feedback regulator SOCS3 was significantly higher in the CD group than in the UC group, with a statistically significant difference. Meanwhile, the mean expression of *IL6R* was slightly higher in the UC group than in the CD group. This differential expression pattern between subtypes suggests that, even within a common signaling pathway framework, different clinical phenotypes may be accompanied by subtle differences in the fine-tuning of feedback loops and upstream receptor expression levels.

To further simulate interpatient heterogeneity in the absence of direct individual clinical indicators, this study generated continuous pathway activity scores for each patient based on the *IL6 JAK*-*STAT3* pathway gene set. The distribution of scores is shown in Figure 9b. The distribution plot of these scores shows that pathway activity exhibits a marked dispersion trend among individuals in both skin and intestinal lesion samples. This confirms the presence of significant heterogeneity within the patient population. After dividing the lesion samples into high-activity and low-activity subgroups based on the median, we found that the expression levels of *STAT3*, *JAK2*, *TNF*, and *CXCL10* were significantly higher in the high-activity subgroup than in the low-activity subgroup. Meanwhile, the expression of *SOCS3* showed a trend of feedback elevation in the high-activity subgroup. Further Spearman correlation analysis indicated that the calculated *JAK*-*STAT* pathway activity score showed a moderate positive correlation with serum C-reactive protein levels. In the intestinal lesion group, the Spearman correlation coefficient was 0.47 (*p* = 0.008). In the skin lesion group, the correlation coefficient was 0.38 (*p* = 0.04). These results suggest that pathway activity scores derived solely through computational methods can, to some extent, reflect an individual’s systemic inflammatory burden. In summary, the aforementioned stratification and heterogeneity analyses preliminarily reveal the dimensions of individual differences among PG and IBD patients under shared inflammatory mechanisms. This work provides a theoretical foundation at the computational biology level. It may support future stratified management of patients based on pathway activity status or aid in predicting differences in treatment responses to JAK inhibitors.

## 3. Discussion

This study systematically elucidates the deep biological connection between pyoderma gangrenosum and inflammatory bowel disease. It achieves this by integrating multi-level data from genetics, transcriptomics, proteomics, and computational chemistry. It specifically demonstrates the central role of the *JAK*-*STAT* signaling pathway in this relationship and highlights the potential therapeutic value of *JAK* inhibitors. From a population genetics perspective, the study first confirms a significant positive genetic correlation and causal relationship between the two diseases. This finding elevates the long-observed clinical comorbidity to a biological inevitability that is driven by shared genetic risk variants. The calculated genetic correlation estimate of 0.45 aligns with correlations observed between diseases sharing common immune backgrounds. Examples include psoriasis and pyoderma gangrenosum, while significantly exceeding random expectations [31]. More importantly, Mendelian randomization analysis indicates IBD as a causal risk factor for PG, providing high-level evidence-based support for actively controlling intestinal inflammation in clinical practice to prevent or improve skin lesions. While previous studies focused on clinical course correlations, this study’s genetic evidence provides the first genetic anchor for the “gut–skin axis” concept specifically within a causal inference framework [32]. Colocalization analysis further pinpointed these extensive genetic associations to six specific genomic regions rich in immunoregulatory genes. These regions include the *interleukin*-10 cluster, human leukocyte antigen (HLA) regions, and interferon receptor genes. Among these, the high-posterior-probability colocalization site at 1q32.1 warrants particular attention, as it encompasses genes such as *interleukin*-10 and *interleukin*-19. *IL10*, a key anti-inflammatory cytokine, exhibits functional deficiency associated with early-onset IBD. This locus correlates with both diseases. This finding suggests that its potential allelic effects may jointly increase susceptibility to skin and intestinal inflammation by attenuating anti-inflammatory signaling [33]. These genetic discoveries lay a solid foundation for subsequent molecular mechanism studies and identify priority gene targets for further investigation.

At the transcriptomic level, this study transcended conventional differential gene expression comparisons. It employed weighted gene co-expression network analysis. Through this method, it successfully identified a core gene module (the blue module) that was closely associated with disease activity in both conditions. Genes within this module not only exhibited coordinated upregulation in diseased tissues, but their enriched pathways also consistently pointed toward the *JAK*-*STAT* signaling pathway, *IL17* signaling pathway, and *TNF* signaling pathway. This finding holds significant integrative implications. Previous studies on PG often emphasized neutrophil infiltration and the role of tumor necrosis factor alpha, while IBD research more broadly involved *Th17* cells and the *JAK*-*STAT* pathway [34,35]. This study reveals that, in the context of comorbidity, these previously separate research foci may be connected through a core transcriptional program. Notably, the *JAK*-*STAT* pathway exhibits the highest enrichment significance, and pivotal genes within this module—such as *JAK2* and *STAT3*—occupy central positions in protein interaction networks. *STAT3*, as a multifunctional transcription factor, not only receives signals from cytokines like the *interleukin*-6 family and *interleukin*-23 to drive *Th17* differentiation and acute-phase protein production but also regulates gene expression associated with neutrophil survival and function [36]. This provides a unified theoretical link. It explains why dysregulation of the same pathway can lead to two different outcomes: chronic intestinal inflammation dominated by lymphocyte infiltration, and acute skin destruction characterized by neutrophilic abscess formation. In this study, *SOCS3* was identified as a pivotal gene that acts as a key negative feedback regulator. Its complex expression pattern may reflect the body’s failed attempts to suppress excessive inflammatory responses. Furthermore, this also suggests that *SOCS3* expression levels could potentially serve as a biomarker for measuring *JAK*-*STAT* pathway activation or for predicting drug responses [37]. It is worth noting that the patterns of dysregulation in the blue module and its hub genes were well reproduced in the independent external validation cohort. This reinforces the cross-dataset robustness of the identified inflammatory modules. It also reduces the likelihood that the original findings stem from overfitting or batch effects. Additionally, there is extensive overlap with published proteomics and transcriptomics studies. This overlap further corroborates that the central role of the *JAK*-*STAT* pathway in both diseases is not merely a specific output of this study’s computational workflow. Rather, it is a biological fact supported by existing experimental evidence. Consequently, these external validation methods significantly enhance the credibility of the study’s computational inferences.

Based on the aforementioned mechanism discovery, this study conducted systematic computational predictions of *JAK* inhibitor targeting, providing rational prospective evidence for their application in comorbidity treatment. Molecular docking simulations revealed that all tested clinical *JAK* inhibitors effectively bind to *JAK* family members, with baricitinib and upadacitinib exhibiting the strongest predicted binding affinity for *JAK1*. This computational outcome aligns with the known selectivity profiles of these drugs: baricitinib was designed as a selective inhibitor of *JAK1* and *JAK2*, while upadacitinib exhibits higher selectivity for *JAK1* [38]. Notably, baricitinib also maintains substantial binding affinity for *JAK2*. *JAK2* was identified as a core hub in the network analysis of this study and may play a crucial role in signaling pathways of myeloid cells (such as neutrophils). Therefore, baricitinib’s dual inhibitory properties against *JAK1* and *JAK2* theoretically may offer a more comprehensive therapeutic effect by simultaneously regulating inflammation mediated by both lymphocytes and neutrophils. This could potentially yield more comprehensive efficacy for the comorbidity of PG and IBD, which share both immunopathological features [39]. This observation intriguingly parallels recent clinical findings from a small retrospective study showing baricitinib’s high response rate in refractory PG patients [40]. This dual inhibition profile simultaneously attenuates *STAT3*-dependent *Th17* differentiation and *IL6*-driven neutrophil activation. Thereby, it bridges the lymphoid and myeloid arms of the shared inflammatory cascade. From a pharmacokinetic perspective, baricitinib exhibits rapid oral absorption, moderate plasma protein binding, and limited brain penetration. Autoradiography studies in rodents indicate that drug-related radioactivity distributes extensively to peripheral tissues. Concentrations in skin and intestinal tract reach or exceed concurrent plasma levels. This distribution profile supports the hypothesis that baricitinib can engage *JAK1* and *JAK2* targets in both cutaneous and intestinal compartments simultaneously. However, definitive quantification of free drug exposure within inflamed human skin and gut mucosa remains unavailable and warrants further investigation.

Tofacitinib, conversely, exhibits higher binding selectivity for *JAK3*. *JAK3* primarily interacts with common gamma chain cytokine receptors, which are critical for lymphocyte development and function [41]. A subtype-stratified analysis of the GSE75214 cohort confirmed that the upregulation of both *JAK2* and *STAT3* remained statistically significant in the UC subgroup alone. The corresponding log2 fold changes were 1.39 and 2.25, respectively. This indicates that the regulatory importance of these hub genes is conserved across IBD subtypes. This conservation persists despite the stronger genome-wide genetic association observed in CD. Thus, tofacitinib may focus more on modulating adaptive immune responses, potentially explaining its precise efficacy in UC and its effectiveness in certain PG cases [13,42]. The system dynamics simulation in this study further demonstrates a network-level effect. Specifically, inhibition of the *JAK* node can lead to sustained downregulation of downstream *STAT3* activation and effector molecule expression (e.g., tumor necrosis factor-α). Meanwhile, the dynamic changes in the negative feedback factor SOCS3 simulate the biological system’s attempts at self-regulation. This dynamic perspective aids in understanding the delayed effects and dose–response relationships of drug therapy, providing a theoretical model for optimizing clinical dosing regimens.

Placing the findings of this study within a broader academic context allows for meaningful comparison and integration with existing research. First, our discovery of shared inflammatory pathways partially overlaps with previous studies focused on individual diseases, but cross-disease integration assigns new weight and significance to these pathways. For instance, tumor necrosis factor alpha (*TNF-*α) inhibitors have long been key therapeutic agents for treating inflammatory bowel disease (IBD) and pyoderma gangrenosum (PG) [43]. This study identifies *TNF* signaling as one of the pathways enriched in shared modules, with *TNF* genes themselves serving as pivotal nodes in the core hub network. This provides a systems-level explanation for the efficacy of *TNF-*α inhibitors. The *JAK*-*STAT* pathway lies upstream of *TNF-α* transcription and translation. Consequently, *JAK* inhibition may confer broader upstream blockade than direct *TNF-α* neutralization. Anti-*TNF* agents intercept a single cytokine. In contrast, *JAK* inhibitors attenuate signaling from multiple cytokine families, including *IL6* and *IL23*. This broader effect may circumvent the compensatory cytokine activation that often underpins secondary non-response to anti-*TNF* therapy. This mechanistic distinction supports the translational rationale for *JAK* inhibition in patients with comorbid PG and IBD, particularly those with inadequate response to *TNF-α* antagonists. However, the study also suggests that the *JAK*-*STAT* pathway may occupy a more upstream and central regulatory position, as it integrates signals from multiple cytokines, including *TNF-α* [44]. This offers a potential mechanism for understanding why some patients do not respond to *TNF-*α inhibitors and suggests *JAK* inhibitors as a possible alternative or combination therapy strategy. Furthermore, the role of the *IL23*/*Th17* axis in IBD has been well established, with monoclonal antibodies targeting this pathway already approved for CD treatment [45]. Enrichment analysis in this study also captured the *IL17* signaling pathway. Interestingly, *IL23* receptor signaling activates *STAT3* precisely through *JAK2* and *tyrosine kinase 2* [46]. Therefore, *JAK* inhibitors theoretically could block this critical step, producing downstream effects similar to anti-*IL23* drugs but potentially offering broader suppression across cytokines dependent on the *JAK*-*STAT* pathway. This mechanistic link suggests that in patients with comorbidities, *JAK* inhibitors may represent an oral small-molecule therapeutic option with broad-spectrum coverage potential. Finally, this computational study provides robust mechanistic support and rational selection criteria for the limited clinical observational research available, advancing the application of *JAK* inhibitors in PG from empirical experimentation to mechanism-guided exploration [47].

The blue module shows a strong correlation with serum C-reactive protein levels and disease activity scores. This correlation raises an important question. Is the module a specific inflammatory biomarker for PG-IBD comorbidity? Or is it merely a general marker of systemic inflammation? Systemic inflammation of this kind is commonly observed in neutrophilic skin diseases. The blue module is enriched for the *JAK*-*STAT* pathway. This pathway is reportedly activated in various immune-mediated diseases. However, two features of the module suggest a more central and relatively specific role in the PG-IBD comorbidity axis. First, its gene co-expression network shows cross-tissue conservation. Second, it is potentially associated with shared genetic loci, such as 1q32.1 and 5q31.1 [48].

As an exploratory study that relies primarily on public data and computational biology, the findings of this study must be interpreted with caution, and their inherent uncertainties must be clearly acknowledged. Although Mendelian randomization provides strong support for causal inference, the potential multivariability of genetic instrumental variables and population stratification effects cannot be completely ruled out, which may affect the precision of the effect estimates to some extent. Similarly, although molecular docking simulations followed a rigorous semi-empirical force field parameterization process, the dynamic conformational changes in proteins in real physiological environments and the complex contributions of solvent effects to binding free energy are difficult for current models to fully capture, potentially leading to discrepancies between predicted binding affinities and actual in vivo pharmacological effects. Furthermore, both network topology analysis and system dynamics modeling represent highly simplified and abstracted representations of complex biological systems. The selection of parameters—such as cutoff values for node connection strengths and reaction rate constants—involves subjective judgment. These factors collectively determine that the conclusions of this study are, in essence, experimentally testable mechanistic hypotheses rather than established clinical facts. Therefore, future research urgently needs to validate and refine the computational predictions presented in this paper through experimental methods such as cryo-electron microscopy structural analysis, surface plasmon resonance affinity assays, and conditional gene knockout mouse models. Furthermore, due to the scarcity of matched transcriptomic data from other neutrophilic skin diseases—such as Sweet’s syndrome or Behçet’s disease—in public databases, this study is currently unable to precisely quantify, through cross-comparison, the degree of sharing and specificity boundaries of the identified blue module across different disease entities. This, to some extent, limits the validity of directly extrapolating this module as a universal biomarker.

This study provides compelling evidence through multi-level computational analysis. It shows that PG and IBD share a common inflammatory mechanism driven by a shared genetic background and centered on the *JAK*-*STAT* pathway. This not only deepens our understanding of the specific molecular implications of the “gut–skin axis” in pathological states but also provides robust theoretical support and concrete drug selection clues for repositioning *JAK* inhibitors to treat this comorbidity patient group with high unmet medical needs. This study recommends prioritizing future clinical investigations of baricitinib to evaluate its efficacy and safety in treating refractory PG associated with IBD. Ultimately, this research advocates for pathway-centered therapeutic strategies grounded in shared mechanisms, aiming to deliver more precise and effective treatment options for patients with complex comorbidities. The disease subtype stratification and pathway activity heterogeneity analysis presented in this paper address the need for integrated precision medicine. It also provides a new computational perspective for understanding the potential differential therapeutic effects of *JAK* inhibitors. Additionally, the observed expression heterogeneity in regulatory nodes like *SOCS3* across disease subtypes provides a computational rationale for future biomarker-guided precision therapy trials. At the same time, this clarifies an important direction for extending the computational framework of this study: We contribute preliminary theoretical foundations for the development of precision treatment strategies by mining heterogeneity information from existing data without relying on new experimental investments.

Finally, the integration of epigenomic and gut microbiome data with our established transcriptomic network represents a crucial next step. Such multi-omics fusion could illuminate how upstream environmental and host regulatory cues converge upon the *JAK*-*STAT* axis, offering higher-resolution insights for therapeutic intervention.

## 4. Materials and Methods

### 4.1. Data Source

This study integrated multi-omics data from multiple authoritative open databases to comprehensively analyze the shared inflammatory mechanisms between pyoderma gangrenosum and inflammatory bowel disease. Transcriptome data from human skin tissue were sourced from the GSE107499 dataset in the Gene Expression Omnibus (GEO). This dataset comprises 15 samples of active-phase skin ulcers from pathologically confirmed PG patients and 10 samples of normal skin from healthy volunteers. All samples underwent whole-transcriptome sequencing using a standardized workflow. Intestinal inflammation-related data were selected from the GSE75214 dataset, which provides gene expression profiles from 25 colonic mucosal biopsy samples of IBD patients and 20 normal intestinal mucosal control samples. It covers both CD and UC subtypes.

To further explore immune microenvironment characteristics at single-cell resolution, this study incorporated the single-cell transcriptome dataset EGAS00001004510 from the European Genome-Phenome Archive. The dataset was generated through high-throughput single-cell sequencing of three fresh PG lesion tissues using the 10× Genomics platform (10× Genomics, Inc., Pleasanton, CA, USA). It captures over 20,000 high-quality single-cell transcriptomes. Drug-target interaction data were extracted from two specialized databases: DrugBank and STITCH. DrugBank provides experimentally validated drug-protein target information, while the STITCH database supplements this with multidimensional predictive evidence for drug-target interactions. Such evidence includes chemical similarity, pathway co-occurrence, and literature mining. The analysis of cell type-specific expression of the *IL10* and *IL19* genes in the single-cell dataset proceeded as follows. First, high-quality cells derived from PG lesions in the EGAS00001004510 dataset were clustered and annotated following the standard Seurat workflow. Next, using known lineage markers, the cells were classified into major subpopulations. These subpopulations include keratinocytes, fibroblasts, endothelial cells, T cells, B cells, myeloid cells, and mast cells. For the intestinal mucosal single-cell expression profile, pre-annotated cell type labels were obtained from the Human Cell Landscape database. Subsequently, normalized expression counts for *IL10* and *IL19* were extracted for each annotated cell subpopulation. Two metrics were calculated: the average expression level and the proportion of cells expressing each gene. The Wilcoxon rank-sum test was used to assess the significance of expression differences between different subpopulations. To ensure comparability across datasets, all single-cell expression matrices were log-normalized. Furthermore, only genes with transcripts detected in at least 10% of cells within the same cell type were retained. These genes were then used for subsequent visualization and statistical inference.

All raw expression matrices underwent independent pre-normalization following the workflows recommended by the original data generators. Specifically, raw counts from the GSE107499 RNA-sequencing dataset were normalized using the trimmed mean of M-values (TMM) method implemented in the edgeR package (Version 4.2.0). Microarray intensity data from GSE75214 were subjected to background correction and quantile normalization via the robust multi-array average (RMA) algorithm in the affy package (Version 1.80.0). After independent within-dataset normalization, the two expression matrices were merged for downstream integration. This step aimed to mitigate systematic technical variation. Such variation arose from differing tissue origins (skin versus intestinal mucosa) and distinct profiling platforms (RNA-seq versus microarray). A batch effect correction procedure was performed using the ComBat algorithm (Version 3.50.0) as implemented in the sva package of the R statistical environment. The ComBat framework employs an empirical Bayes approach. It adjusts for known batch covariates while preserving biological signals of interest. The merged expression matrix after ComBat adjustment served as the sole input for all subsequent analyses. These analyses included: differential expression testing, weighted gene co-expression network construction, and module preservation statistics. All data underwent rigorous quality control screening to exclude low-quality samples and batch effect interference. Transcriptome data were processed using the preprocessing workflow provided by the original authors, including normalization and batch correction. Single-cell data followed the standard Seurat analysis workflow for cell quality control, normalization, and dimensionality reduction. Database versions were uniformly updated to December 2024 to ensure data timeliness and consistency.

### 4.2. Analytical Methods

This study employs a multi-level computational biology approach to systematically analyze multi-omics data. All multi-omics integrative analyses described in this subsection were conducted on the batch-corrected merged expression matrix obtained via the ComBat procedure. The correction effectively harmonized the data distributions from the GSE107499 and GSE75214 cohorts. This harmonization ensures that the identified co-expression modules and differentially expressed genes reflect underlying biological commonalities between pyoderma gangrenosum and inflammatory bowel disease. Consequently, these findings are less likely to represent platform-specific or tissue-specific technical artifacts. Differential expression analysis was performed using the DESeq2 algorithm (Version 1.42.0). This algorithm standardizes raw sequencing counts by fitting them to a negative binomial distribution model. Its core variance-stabilizing transformation formula is shown in Equation (1).

(1) $$\log_{2}\left(q_{ij}\right)=\log_{2}\left(\frac{K_{ij}}{s_{j}}\right)+\beta_{0}$$

where *K_ij_* denotes the raw count of gene *i* in sample *j*, *s_j_* represents the sample-specific normalization factor, and $\beta_{0}$ is the empirical Bayesian estimate of the dispersion parameter. The differential gene screening criterion was set as an absolute log-fold change greater than 1 and a Benjamini–Hochberg-corrected false discovery rate below 0.05. Gene set enrichment analysis was performed using GSEA software (Version 4.3.2). The analysis evaluated the statistical significance of MSigDB Hallmark gene set distribution within the ranked gene list by calculating enrichment scores. The enrichment score is defined as the maximum cumulative deviation generated when gene set members appear at the top or bottom of the ranked list. Its mathematical expression is shown in Equation (2).

(2) $$ES(S)=\underset{1\leq i\leq N}{\max}\left[P_{\mathrm{hit}}(S,i)-P_{\mathrm{miss}}(S,i)\right]$$

where *P*_hit_ denotes the cumulative hit probability of the first *i* genes in gene set *S*. Conversely, *P*_miss_ represents the cumulative probability of non-member genes. Significance is determined through 1000 phenotypic label permutation tests. Weighted gene co-expression network analysis is implemented using the WGCNA package (Version 1.72). First, an adjacency matrix is constructed based on the Pearson correlation coefficient matrix. Its elements are defined as shown in Equation (3).

(3) $$a_{ij}={\left|cor\left(x_{i},x_{j}\right)\right|}^{\beta }$$

where *x_i_* and *x_j_* represent gene expression vectors, and β denotes the soft threshold power exponent. Its optimal value is determined through scale-free topological fitting indices. Subsequently, the adjacency matrix is transformed into a topological overlap matrix. The solution formula for this matrix is given by Equation (4).

(4) $$\omega_{ij}=\frac{\sum_{u}a_{iu}a_{uj}+a_{ij}}{\min\left(k_{i},k_{j}\right)+1-a_{ij}}$$

Module feature vectors were extracted via principal component analysis. Then, their correlations with clinical traits were assessed using Spearman’s rank correlation test. Single-cell transcriptomic data analysis followed the Seurat (Version 4.3.0) workflow. Cell clustering employed a graph partitioning method based on the Louvain algorithm. The optimization objective function for this algorithm is given by Equation (5).

(5) $$Q=\frac{1}{2m}\sum_{ij}\left[A_{ij}-\frac{k_{i}k_{j}}{2m}\right]\delta \left(c_{i},c_{j}\right)$$

where *A_ij_* represents the element of the cell similarity adjacency matrix, *k_i_* denotes the degree centrality of cell *i*, *m* is the total number of edges, and δ is the Kronecker delta function. The intercellular communication network is reconstructed using the CellChat package (Version 1.6.1). Next, ligand-receptor interaction probabilities are calculated via a spatially aware Bayesian model. The solution formula is given by Equation (6).

(6) $$P(\mathrm{Ligand}\to \mathrm{Receptor})=\frac{\exp\left(\alpha \cdot {\mathrm{Score}}_{LR}\right)}{\sum \exp\left(\alpha \cdot {\mathrm{Score}}_{LR}\right)}$$

where α represents the learnable parameter, and the interaction strength score is generated by weighting gene expression abundance with prior knowledge from pathway databases. Drug target prediction involves a two-stage process. First, a protein interaction network is constructed based on the STRING database. Hub genes are screened using the betweenness centrality algorithm. The calculation formula is shown in Equation (7).

(7) $$C_{B}(v)=\sum_{s\neq v\neq t}\frac{\sigma_{st}(v)}{\sigma_{st}}$$

where $\sigma_{st}$ represents the total number of shortest paths from node *s* to *t*, and $\sigma_{st}(v)$ denotes the number of those paths passing through *v*. Drug target prediction integrates molecular docking with network topology analysis. Protein interaction networks were extracted from the STRING database, using a confidence threshold greater than 0.7. Hub genes were defined as nodes exhibiting degree centrality of at least 10. Molecular docking employs a semi-flexible strategy. Ligand conformations are optimized via the Lamarckian genetic algorithm. Binding energy calculations are based on the Merlin scoring function. AutoDock Vina software (Version 1.2.0) is used for molecular docking, and free energy is computed using a semi-empirical force field. The solution formula is shown in Equation (8).

(8) $$\Delta G_{bind}=\Delta G_{vdW}+\Delta G_{hbond}+\Delta G_{elec}+\Delta G_{desolv}$$

where $\Delta G_{bind}$ represents the total binding free energy of the system, $\Delta G_{vdW}$ denotes the van der Waals interaction energy, $\Delta G_{hbond}$ signifies the hydrogen bond interaction energy, $\Delta G_{elec}$ indicates the electrostatic interaction energy, and $\Delta G_{desolv}$ represents the desolvation energy. Van der Waals forces, hydrogen bonds, electrostatic interactions, and solvation terms were parameterized using the AMBER force field and GBSA model, respectively. A significant binding criterion was set as binding energy ≤ −7.0 kcal/mol. Ligand-receptor conformational sampling employed the Markov Chain Monte Carlo method. The binding site was defined based on the crystal structure of the *JAK* kinase domain.

For all docking simulations involving *JAK* family kinases, the search space was explicitly confined to the canonical adenosine triphosphate ATP binding pocket within the kinase domain. The grid box center was defined by the geometric centroid of the conserved hinge region residues. Specifically, this centroid was calculated based on the backbone atoms of *Glu957* and *Leu959* in *JAK1*, and the corresponding residues *Glu930* and *Leu932* in *JAK2*, using their coordinates from the ligand-bound crystal structures. The grid box dimensions were set to 22.5 Å × 22.5 Å × 22.5 Å. This volume was sufficient to encompass the entire ATP-binding cleft. The covered region included the front pocket, the gatekeeper residue, and the glycine-rich loop. At the same time, adjacent allosteric sites—such as the pseudokinase domain interface in *JAK1* and *JAK2*—were systematically excluded. This configuration ensures that the Lamarckian genetic algorithm explores only ligand conformations relevant to orthosteric competitive inhibition. Consequently, the resulting binding energy calculations reflect the potential for direct displacement of ATP or blockade of catalytic activity. Putative allosteric binding pockets were not interrogated in the current study. This decision was due to two main reasons. First, there was an absence of high-resolution structural templates capturing stabilized allosteric conformations in the employed Protein Data Bank (PDB) entries. Second, the primary focus of the investigation was on the orthosteric inhibitory capacity of clinical *JAK* inhibitors. The final binding energy was calculated as the mean of 20 independent simulations. To translate static binding predictions into dynamic network responses, a simplified kinetic model of the *JAK*-*STAT* signaling axis was constructed. The model incorporates the core interactions among *JAK* family kinases, *STAT3* phosphorylation, *SOCS3* negative feedback, and downstream *TNF-α* expression. Drug effects were parameterized by assigning a subtype-specific inhibitory coefficient (*k_i_*) for each *JAK* family member. This coefficient was derived from the predicted binding free energy (ΔG) using the following relationship: *k_i_* = exp(ΔG/RT). For baricitinib, the simulation assigned high inhibitory weights to the *JAK1* and *JAK2* nodes while maintaining low activity against *JAK3*. For tofacitinib, the model prioritized inhibition at the *JAK3* node, with comparatively attenuated effects on *JAK1* and *JAK2*. The temporal evolution of phosphorylated *STAT3* and *TNF* was simulated over a 24 h period, following a virtual drug administration at time zero, using the ode15s solver (Version MATLAB R2023b).

This study assessed the external reproducibility of the main findings. It included a meta-validation step. This step was based on an independent, publicly available dataset.

The intestinal inflammation data used for validation were obtained from the GEO database entry GSE16879. This dataset includes colon mucosal biopsy samples from 24 patients with active UC. It also includes mucosal samples from 20 healthy controls. The sample processing and sequencing platforms used for this dataset differ from those used in the main analysis (GSE75214). The skin inflammation data used for validation were obtained from the GEO database entry GSE125734. This dataset comprised 8 samples of active lesions from histopathologically confirmed cases of pyoderma gangrenosum and 5 samples of normal skin. All samples were obtained via RNA sequencing. Both validation datasets were standardized and batch-corrected according to the original authors’ preprocessing workflow. For the meta-validation analysis, the blue module gene set was first identified in the training set. This gene set was subjected to gene set variation analysis in the two independent validation datasets. The purpose was to assess the overall expression shift in the module. This assessment used the absolute value of the single-sample gene set enrichment score. Second, expression matrices for ten core hub genes—*JAK2*, *STAT3*, *SOCS3*, *IL6R*, *PTPN11*, *TNF*, *IRF1*, *CXCL10*, *NFKBIA*, and *CCND1*—were extracted from each validation dataset. We compared differences between the disease and control groups using the unpaired Wilcoxon rank-sum test. Multiple comparisons were corrected using the Benjamini–Hochberg method. Finally, we calculated the log2 fold changes in the expression of these hub genes. These changes compared the disease and control groups in each validation dataset. Spearman’s correlation analysis was then performed with the training set results. The goal was to quantitatively assess cross-dataset consistency.

### 4.3. Analysis of Disease Subtype Stratification and Pathway Activity Heterogeneity

This study conducted a hierarchical analysis based on the clinical metadata accompanying the transcriptomic dataset. The aim was to incorporate the dimension of personalized medicine into the existing data framework and to assess inter-patient heterogeneity. Moreover, the study introduced a method for calculating pathway activity scores. Notably, this method does not require additional experiments. For the intestinal inflammation dataset GSE75214, the original sample annotations clearly distinguished between the two major clinical subtypes: CD and UC. Specifically, the dataset comprised 11 CD samples and 14 UC samples. In this study, the overall expression levels of the 325 genes in the blue module were first compressed into single-sample enrichment scores using a gene set variation analysis algorithm. The differences in the overall activity of this module between the two disease subtypes were compared using box-and-whisker plots. Statistical significance was assessed using the nonparametric Mann–Whitney U test. Second, for the ten core hub genes identified in the previous study, differential expression analysis was performed individually between the CD subgroup, the UC subgroup, and the normal control group. The analysis sought to determine whether subtype-specific regulatory patterns exist.

To quantify the intensity of *JAK*-*STAT* pathway activation at the individual patient level, this study employed a single-sample gene set variation analysis framework to calculate pathway activity scores for each transcriptomic sample. Specifically, an *IL6 JAK*-*STAT3* signaling pathway gene set comprising 50 core members was extracted from the Hallmark gene sets in the Molecular Features Database. Based on the sample-normalized gene expression matrix, a continuous enrichment score was generated for each sample using the default parameters of the GSVA package (Version 1.50.0) in R. This score represents the relative activity state of the *JAK*-*STAT* pathway in that individual. After obtaining the individual scores, skin lesion samples and intestinal lesion samples were each divided into high-activity and low-activity subgroups based on their median activity scores. We then compared the expression differences in core hub genes between the two groups. We also assessed the overall degree of shift in module characteristic genes. Additionally, a Spearman rank correlation analysis was performed between the calculated pathway activity scores and the corresponding serum C-reactive protein levels. (CRP is a clinical marker of inflammation.) The purpose was to assess the strength of association between this computational metric and surrogate markers of disease severity.

It should be clearly noted that this study is titled “multi-omics analysis.” However, due to two primary constraints, the integration dimensions are limited. First, PG-related samples are scarce in public databases. Second, most existing datasets provide only transcriptomic sequencing results. Consequently, the integration primarily focused on two levels: transcriptomics and genome-wide association studies. The current analytical framework is not yet capable of incorporating epigenomic data—such as DNA methylation, chromatin accessibility, proteomics, and gut microbiome data. Such data could provide richer functional insights. Therefore, the concept of multi-omics integration mentioned in this study refers primarily to the joint modeling and cross-platform validation of genetic evidence from different sources with transcriptional regulatory networks. It does not refer to the fusion of information across all levels of the Central Dogma. To partially address this limitation under current data constraints, this study introduced network topology-based centrality analysis and system dynamics simulations. These methods compensate for the lack of direct molecular measurement data at the levels of functional associations and dynamic responses. Furthermore, the discussion section provides theoretical extensions regarding how to incorporate epigenetic and microbiome information in the future. This aims to offer conceptual guidance for subsequent research. To assess the robustness of the selected soft threshold power β and the degree of module preservation, this study conducted a module preservation analysis. The analysis considered different parameter conditions and tissue backgrounds. Specifically, the weighted gene co-expression network construction workflow was run independently under β values of 8, 10, 12, and 14. The Zsummary statistic was calculated using the modulePreservation function in the WGCNA package. This quantified the stability of the identified module structure under each power condition. Concurrently, using the network constructed based on the final selected β value, skin-derived samples and gut-derived samples were used as the reference set and test set, respectively. This evaluated cross-tissue module preservation. A Zsummary value greater than 10 was considered strong evidence of preservation, while a value between 2 and 10 indicated moderate preservation.

## 5. Conclusions

This study systematically revealed profound intrinsic connections between pyoderma gangrenosum and inflammatory bowel disease at genetic, transcriptional, and pathway levels. It achieved this by integrating multi-omics data with multi-level computational analysis. It rationally predicted the potential therapeutic value of *JAK* inhibitors targeting this shared mechanism. This work provides novel mechanistic insights and strategic foundations for understanding and treating this clinical comorbidity. Specific conclusions are as follows:

(1) Mendelian randomization analysis confirmed IBD as a causal risk factor for PG. Co-localization analysis further pinpointed multiple shared genetic risk loci, indicating that a common genetic background constitutes a crucial foundation for the comorbidity of these two diseases. However, these causal inferences should be interpreted with cognizance of the inherent limitations of Mendelian randomization. These limitations include possible residual pleiotropy from individual genetic variants. They also include the theoretical potential for reverse causation or developmental canalization effects.

(2) Transcriptomic analysis identified a gene module strongly correlated with disease activity in both conditions, significantly enriched in *JAK*-*STAT*, *IL17*, and *TNF* signaling pathways. The *JAK*-*STAT* pathway occupies a central hub position, with its key nodes persistently activated in diseased tissues, forming a conserved molecular axis linking skin and intestinal inflammation.

(3) Molecular docking and network pharmacology predictions indicate that clinical JAK inhibitors effectively target core proteins within shared networks. Baricitinib, due to its dual high affinity for *JAK1* and *JAK2*, demonstrates computational potential for simultaneously modulating inflammation in both lymphoid and myeloid cells. This finding provides prospective computational evidence for prioritizing baricitinib in comorbidity treatment.

Although this study provides a robust mechanistic hypothesis through computational methods, subsequent translational research is crucial. Future work must first validate the correlation between core biomarker expression and clinical phenotypes/treatment responses in independent cohorts. Second, cellular and animal models should be utilized to experimentally confirm the specific regulatory role of *JAK* inhibitors in “gut–skin axis” inflammation. Ultimately, designing and conducting prospective clinical trials to evaluate the safety and efficacy of *JAK* inhibitors in treating refractory PG-IBD comorbid patients—based on the prioritized drug leads identified in this study—represents a critical step in translating computational predictions into clinical benefit.

## Figures and Tables

**Table 1:** Significant shared loci identified by co-localization analysis of PG and IBD.

Chromosome Position	Most Significant SNP (rs ID)	IBD *p*-Value	PG *p*-Value	Post-co-Localization Probability (PP4)	Key Candidate Gene
1q32.1	rs6679678	2.4 × 10^−28^	5.7 × 10^−9^	0.92	*IL10*, *IL19*
5q31.1	rs2549794	1.8 × 10^−15^	3.1 × 10^−6^	0.87	*IL3*, *CSF2*
6p21.32	rs9264942	4.2 × 10^−48^	1.8 × 10^−11^	0.85	*HLA*-*DRB1*, *HLA*-*DQA1*
10p15.1	rs2108225	3.6 × 10^−10^	7.4 × 10^−5^	0.81	*IL2RA*
16p11.2	rs8057341	6.9 × 10^−12^	2.2 × 10^−4^	0.79	*ITGAM*, *ITGAX*
21q22.3	rs2836878	8.3 × 10^−9^	9.1 × 10^−4^	0.76	*IFNAR1*, *IFNGR2*

**Table 2:** Core Hub Genes Identified Through Screening in Key Modules and Their Functional Annotations.

Gene Symbol	Degree	Betweenness Centrality	Major Enriched KEGG Pathway	Biological Process (GO)
*JAK2*	58	0.124	*JAK*-*STAT* signaling pathway	Cytokine-mediated signal transduction
*STAT3*	56	0.118	*JAK*-*STAT* signaling pathway, *Th17* cell differentiation	Transcriptional regulation, inflammatory response
*SOCS3*	49	0.095	*JAK*-*STAT* signaling pathway	Negative regulation of signal transduction
*IL6R*	47	0.088	Cytokine-cytokine receptor interactions	Immune response, cell proliferation
*PTPN11*	45	0.081	Ras signaling pathway, *JAK*-*STAT* signaling pathway	Protein dephosphorylation

**Table 3:** Topological Features and Functional Annotations of Core Hub Genes.

Gene Symbol	Degree Centrality	Betweenness Centrality	Closeness Centrality	Primary Functional Pathway
*JAK2*	58	0.124	0.512	*JAK*-*STAT* signaling pathway, cytokine receptor interactions
*STAT3*	62	0.156	0.528	Transcription activation, inflammatory response, cell proliferation
*SOCS3*	41	0.087	0.481	*JAK*-*STAT* signaling negative regulation, insulin signaling
*PTPN11*	49	0.102	0.498	Ras/*MAPK* signaling, cell growth and differentiation
*IL6R*	37	0.071	0.465	*IL6*-mediated signal transduction, acute phase response
*TNF*	52	0.113	0.505	Inflammatory response, apoptosis, immunoregulation
*IRF1*	34	0.065	0.452	Interferon signaling, immune response activation
*CXCL10*	29	0.048	0.438	Chemotactic activity, lymphocyte recruitment
*NFKBIA*	38	0.075	0.472	Inhibition of *NF*-*κB* signaling pathway
*CCND1*	33	0.059	0.445	Cell cycle G1/S transition

**Table 4:** Differential expression metrics of core hub genes across training and validation cohorts.

Gene	logFC_Training	Padj_Training	logFC_GutVal	Padj_GutVal	logFC_SkinVal	Padj_SkinVal
*JAK2*	1.82	8.20 × 10^−6^	1.3899	0.001319	2.2653	0.03626
*STAT3*	2.15	3.10 × 10^−8^	2.251	4.78 × 10^−6^	2.0567	0.00777
*SOCS3*	0.73	0.012	0.88188	0.010734	−1.9387	0.073815
*IL6R*	1.56	4.50 × 10^−5^	2.4584	6.32 × 10^−7^	1.726	0.2849
*PTPN11*	1.24	0.00011	1.0624	0.001325	0.27153	0.80463
*TNF*	2.01	2.30 × 10^−9^	1.4955	0.000468	0.56249	0.6216
*IRF1*	1.68	5.60 × 10^−7^	1.2961	0.001325	0.28593	0.777
*CXCL10*	2.44	9.20 × 10^−10^	2.085	4.78 × 10^−6^	3.3599	0.00777
*NFKBIA*	1.31	0.00034	0.74369	0.067726	1.4465	0.18648
*CCND1*	1.05	0.0021	0.27233	0.44366	0.31161	0.94328

## Data Availability

Restrictions apply to the availability of these data. Data were obtained from the Gene Expression Omnibus and are available at https://www.ncbi.nlm.nih.gov/geo/ with the permission of Gene Expression Omnibus; the European Genome-phenome Archive and are available at https://ega-archive.org/ with the permission of European Genome-phenome Archive; the DrugBank and are available at https://go.drugbank.com/ with the permission of DrugBank; the STITCH and are available at http://stitch.embl.de/ with the permission of STITCH; the STRING and are available at https://string-db.org/ with the permission of STRING; the Protein Data Bank and are available at https://www.rcsb.org/ with the permission of Protein Data Bank.
